# Nasal Carriage Rate, Antimicrobial Susceptibility Pattern, and Associated Factors of* Staphylococcus aureus* with Special Emphasis on MRSA among Urban and Rural Elementary School Children in Gondar, Northwest Ethiopia: A Comparative Cross-Sectional Study

**DOI:** 10.1155/2018/9364757

**Published:** 2018-12-11

**Authors:** Abiye Tigabu, Moges Tiruneh, Feleke Mekonnen

**Affiliations:** ^1^Department of Medical Microbiology, School of Biomedical and Laboratory Sciences, University of Gondar, Gondar, Ethiopia; ^2^Department of Medical Laboratory Sciences, School of Health Sciences, College of Medicine and Health Sciences, Bahir Dar University, Bahir Dar, Ethiopia

## Abstract

**Introduction:**

* Staphylococcus aureus* is a Gram-positive, catalase-positive, and coagulase-positive bacterial species commonly found on the skin and in the nose of most healthy individuals. The anterior nares of nose are the most frequent carriage sites for* S. aureus* in both adults and children. Methicillin resistance among* S. aureus* isolates has steadily increased worldwide.

**Objective:**

The main objective of this study was to determine nasal carriage rate, antimicrobial susceptibility pattern, and associated risk factors of* Staphylococcus aureus* with special emphasis on MRSA among urban and rural elementary school children in Gondar, Northwest Ethiopia.

**Method:**

A community based comparative cross-sectional study was conducted on 622 urban and rural elementary school children in Gondar from January 1^st^ to March 30^th^, 2018. Data was collected using a questionnaire and nasal swab samples were collected by sterile cotton tip swab moistened with sterile normal saline. Collected samples were inoculated on mannitol salt agar and incubated aerobically at 37°C for 24 hrs.* S. aureus* was confirmed by observing colony characteristics and biochemical tests. MRSA was detected using cefoxitin disc by Modified Kirby-Bauer disk diffusion technique. Finally data was entered, cleared, and checked using Epi-info version 7 and exported to SPSS version 20 for analysis. Odds ratio and logistic regression were used for statistical association. P-value ≤ 0.05 at 95% CI was considered for statistical association.

**Result:**

Of the 622 school children, the overall prevalence of* S. aureus* was 143/622 (23%). Of them, 14/143 (9.79%) were MRSA. The carriage rate in urban schools was 83/622 (13.3%) whereas it was 60/622 (9.6%) in rural schools. The prevalence of MRSA among urban schools, 9.1%, was higher than their urban counterparts, 0.7%. Gentamycin, clindamycin, and ciprofloxacin were the most effective whereas penicillin and tetracycline were resistant. Children's fathers' educational status and number of children in class room were significantly associated with* S. aureus* but only living in urban of children significantly associated with MRSA.

**Conclusion:**

This study showed high prevalence of* S. aureus* and MRSA, 143/622 (23%) and 14/143 (9.79%), respectively. So, decolonization of nasal carriers of MRSA and reducing the number of students per classroom should be addressed. Moreover, regular large scale survey should be conducted to assess the burden and intervene accordingly.

## 1. Introduction

*Staphylococcus aureus* (*S. aureus*) is a Gram-positive, catalase-positive, and coagulase-positive bacteria commonly found mainly on the skin and nasal mucosa of most healthy individuals [1]. It is a frequent cause of clinically important infections ranging in severity from superficial infections to severe invasive diseases [2]. Approximately 20% of individuals are persistently nasal carriers of* S. aureus* and the rest 30% are intermittently colonized [3]. The anterior nares of nose are the primary reservoir for replication and spread to other body sites [4, 5].

*Staphylococcus aureus* exhibits increasing virulence and resistance to various antibiotics, complicating prevention, and treatment of infections [6]. Virulence factors are tightly regulated and act to degrade host cells, tissues, change the immune response, and enable for the dissemination inside and outside the host cell [7]. Overuse and misuse of antibiotics led to the emergence of bacterial antibiotic resistance [8]. Methicillin-resistant* S. aureus* (MRSA) carriage in healthy children is a major asymptomatic reservoir with an ability to quickly spread of MRSA within the community [9, 10].

MRSA is resistant to a large group of antibiotics called the beta lactams. Methicillin is a *β*-lactam antibiotic produced to treat penicillin resistant* S. aureus*. Methicillin-resistant* S. aureus* is a pathogenic strain responsible for difficult to treat infections in human [11]. MRSA resistance to antibiotics encoded by the mobile genetic element Staphylococcal chromosomal cassette (SCC) which carry the mecA gene and these elements vary in size and genetic content. The mecA gene encodes an altered penicillin binding protein (PBP2a) which permits the bacteria* S. aureus* to grow in the presence of methicillin and other *β*-lactam antibiotics [12, 13]. PBP2a is located in the bacterial cell wall and has a low binding affinity for *β*-lactam antibiotics [14].

In Ethiopia, no population based study had been carried out on nasal carriage rate, antibiotic susceptibility pattern, and associated risk factors of* S. aureus* and MRSA among school children particularly in Gondar, Northwest Ethiopia. Thus, this study is intended to assess and fill the information gap of the current nasal carriage rate, antibiotic susceptibility pattern, and associated risk factors of* S. aureus* and MRSA among elementary school children in Gondar town. Also to indicate the prevention and control measures in the general community, it will also be used as preliminary information for future studies.

## 2. Materials and Methods

### 2.1. Study Area

The study was conducted in five governmental urban elementary schools in Gondar town and five governmental rural elementary schools surrounding Gondar town; Gondar town is the capital of North Gondar administrative zone, in Amhara region, Northwest Ethiopia.

### 2.2. Study Design and Periods

A community-based comparative cross-sectional study was conducted to determine nasal carriage rate, antimicrobial susceptibility pattern, and associated factors of* Staphylococcus aureus* with special emphasis on MRSA among elementary school children from January 1^st^ to May 30^th^, 2018.

### 2.3. Study Population

School children from recruited schools who gave sociodemographic information and nasal swab samples were included whereas children who were unable to give sociodemographic information, nasal swab specimen, and those who were on antibiotic therapy at the time of data collection were excluded

### 2.4. Inclusion and Exclusion Criteria

#### 2.4.1. Inclusion Criteria

All randomly selected school children from recruited schools who have agreed to give sociodemographic information and nasal swab samples were included.

#### 2.4.2. Exclusion Criteria

Children who were unable to give sociodemographic information and nasal swab specimen and those who were involuntary to participate in the study due to different reasons and children who were on antibiotic therapy at the time of data collection were also excluded.

### 2.5. Sample Size Determination and Sampling Technique

Single population proportion formula was used to determine the sample size and the calculated sample size was 258. However, adding 20% nonresponse rate and a design effect of two, the final calculated sample size was 622. A multistage sampling technique was used to select schools by using simple random sampling technique and stratifying the schools to grades and sections. The number of study participants were allocated proportionally to each schools and grades based on the school sampling frame and the study subjects were selected by simple random sampling technique (lottery method).

### 2.6. Sociodemographic Data and Nasal Swab Specimen Collection

A pretested questionnaire based on postulated or known risk factors were developed and modified to explore the objectives of the study. Then sociodemographic characteristics and other relevant information were collected. Nasal swab specimen were collected by using sterile cotton tip swabs prewetted with sterile saline for each anterior nares by inserting the swab and gently rotating four to five times both in clockwise and in anticlockwise direction from each study participants. The nasal swabs were collected and inoculated immediately in a properly labeled sterile Tryptone Soya Broth (TSY) (Oxoid Ltd. England) and transported by using vaccine carrier which has ice box to maintain the temperature at 2-8°C until it reaches the laboratory.

### 2.7. Laboratory Inoculation and Identification

Each nasal sample was inoculated onto mannitol salt agar (Oxoid Ltd. England) and the plate was incubated aerobically at 37°C for 24 hrs. The sample that was positive for mannitol fermentation and golden yellow colonies on mannitol salt agar was further inoculated on blood agar (Oxoid Ltd. England) plate and incubated at 37°C for 24 hrs. The isolates obtained were identified using standard microbiological methods including colony morphology, Gram's stain reaction, and biochemical tests such as catalase and coagulase. Finally isolates that were golden yellow colony on mannitol salt agar and blood agar pate, Gram-positive cocci in clusters, catalase, and coagulase positive were confirmed as* S. aureus*.

### 2.8. Antimicrobial Susceptibility Testing

A suspension of pure colony from each confirmed culture isolate was done in sterile normal saline and incubated at 37°C for at least 15 minutes. The suspension was adjusted at 0.5% MacFarland standard. Then Modified Kirby-Bauer disk diffusion technique was implemented for antibiotic susceptibility pattern using different antibiotics such as penicillin (10*μ*g), erythromycin (15*μ*g), clindamycin (2*μ*g), gentamicin (10*μ*g), cotrimoxazole (1.25/23.75*μ*g), cefoxitin (30 *μ*g), ciprofloxacin (30 *μ*g), and tetracycline (30 *μ*g). Moreover, inducible clindamycin resistance was tested using D-test by putting erythromycin and clindamycin discs at a distance of 15 mm from each other on Muller Hinton agar plate (Oxoid Ltd. England). Then after incubation at 37°C for 24 hrs, flattening of zone (D-shaped) around clindamycin in the area between erythromycin and clindamycin discs was observed and interpreted as inducible clindamycin resistant.

All* Staphylococcus aureus* isolates were tested for methicillin susceptibility patterns by using Modified Kirby-Bauer disc diffusion technique. Cefoxitin (30*μ*g) (Oxoid Ltd. England) discs were placed in the plates and then incubated aerobically at 35°C for 24 hrs. Zone inhibition in millimeters was measured with a ruler. Isolates were classified as resistant, intermediate, and sensitive based on* CLSI 2017* interpretation [15]. Finally those which were resistant to cefoxitin (≤ 21 mm) were confirmed as MRSA. Known strains of* S. aureus* (ATCC 25923) was inoculated to cheek the performance of culture media as a positive control for catalase, coagulase, and antibiotic susceptibility testing. And* Escherichia coli* (ATCC 25922) strain was used as a negative control. Gram stain reagents were also cheeked by preparing smears from known* S. aureus* (ATCC 25923) strain and observing them under the microscope.

### 2.9. Data Entry and Analysis

Data was entered using EPI-Info version 7 and its completeness and clearance was checked and then transferred to SPSS version 20 for analysis. The characteristics of the study population were summarized using frequencies, mean, and standard deviation. Binary logistic regression was done to determine the association of variables with* S. aureus* and MRSA. Crude odds ratio was calculated. Moreover, adjusted odds ratio was computed using multivariate logistic regression for variables with p value ≤ 0.2. P-value ≤0.05 at 95% CI was considered as statistically significant.

## 3. Result

### 3.1. Sociodemographic Characteristics

A total of 622 urban and rural elementary school children were included in the study; of these, 317/622 (51%) were males. The ages of the study participants ranged from 6 to 25 years with a mean age of 11.9 years (SD+2.9). Most of the study participants, 345/622 (55.5%), belonged to the age group of 11-15 years of age and 345/622 (55.5%) were from urban elementary schools while the rest, 277/622 (44.5%), were from rural schools. Among the study participants, 321/622 (51.6%) had a family size of greater than five members and 271/622 (43.6%) had mothers who were unable to read and write (Table 1).

Majority of the study participants, 545/622(87.6%), had no history of chronic disease, but from the total of participants, almost half, 299/622(48.1%), of the study participants had history of visit to hospitals/clinics. On top of that, 522/622(83.9%) had not history of hospitalization; 583/622 (93.7%) had not history of surgery; 551/622 (88.6%) had not history of contact with health care worker; 529/622(85%) had not history of antibiotic usage in the past 4 weeks; and the rest, 531/622 (85.4%), had not history of respiratory infection (Table 1).

Majority, 295/622 (47.4%), of the study participant's family's average monthly income was less than 18 dollar followed by 197/622 (31.7%), greater than 36 dollar, and 130/622 (20.9%), 18-36 dollar incomers. Majority of the fathers of the study participants, 211/622 (33.9%), were unable to read and write followed by 146/622 (23.5%), primary school; 103/622 (16.6%), secondary school; 97/622 (15.6%), informal education; and 65/622 (10.5%), above grade 12 attenders (Table 1).

### 3.2. Prevalence of* Staphylococcus aureus *and MRSA

Out of 622 study participants, the overall prevalence of* S. aureus* was 143/622 (23%); of these 14/143 (9.79%) were MRSA strains. From the total of 143 isolates, 79/622(12.7%) were isolated from male participants, of which 7/79 (8.86%) were MRSA. And 64/622 (10.3%) isolates were from females, of which 7/64 (10.94%) were MRSA isolates. Highest nasal carriage rate of* S. aureus*, 26/622 (4.2%), was shown among grade four students, from which MRSA stains were 2/26 (7.7%) (Table 2).

In urban elementary schools, the* S. aureus* carriage rate was 83/622 (13.3%) whereas in the rural schools, it was 60/622 (9.6%). The frequency of MRSA among* S. aureus* positive children in urban and rural school children was 13/143 (9.1%) and 1/143 (0.7%), respectively. The overall prevalence of inducible clindamycin resistant* S. aureus* in this study was 12/622 (1.93%).

The frequency of* S. aureus* nasal carriage in males was 45/622(7.23%) in urban as compared to 34/622 (5.5%) in rural schools and that of females was 38/622 (6.1%) in urban as compared to 26/622 (4.2%) in rural schools. The prevalence of MRSA nasal carriage in males was 6/143 (4.2%) in urban as compared to 1/143 (0.7%) in rural schools while that in females was 7/143(4.9%) in urban as compared to 0/143(0%) in rural schools. The carriage rate of* S. aureus* reached its peak at the age of 11, 20/622 (3.2%). High prevalence of* S. aureus* nasal carriage, 81/622 (13%), was observed under age group of 11-15 years of age. But high carriage rate of MRSA, 8/143 (5.6%), was observed under age group of 6-10 years of age (Table 2).

**Figure 1:**
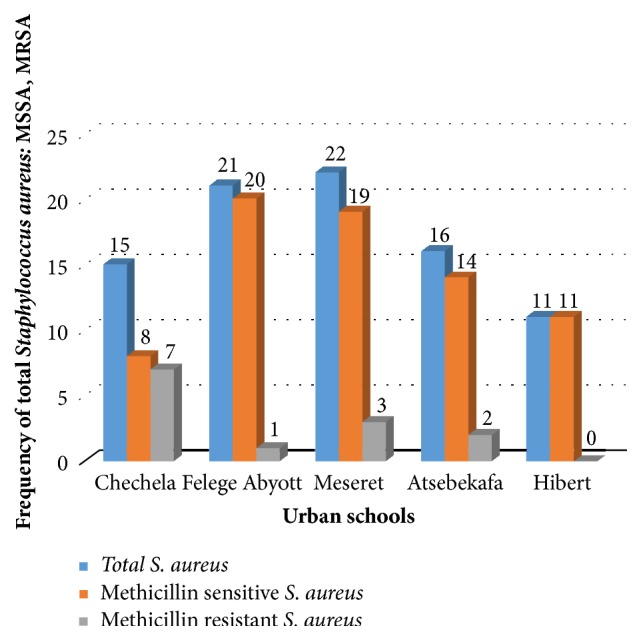
Frequency of total* Staphylococcus aureus*, MSSA, and MRSA in five urban elementary school children, Gondar town, Northwest Ethiopia, 2018.

**Figure 2:**
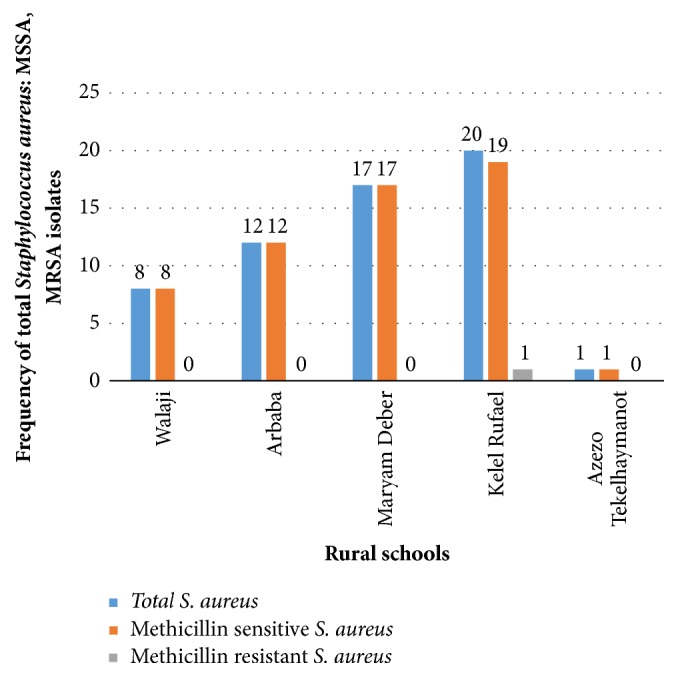
Frequency of total* Staphylococcus aureus*, MSSA, and MRSA in five rural elementary school children, surrounding Gondar town, Northwest Ethiopia, 2018.

Among urban elementary school children, high rate of* S. aureus* isolates was observed in Meseret, 22/343 (6.4%), followed by Felege Abyot, 21/343 (6.1%); Atsebekafa, 16/343 (4.7%); Chechela, 15/343 (4.4%); and Hibret, 11/343 (3.2%), while high rate of MRSA isolation was observed in Chechela, 7/85 (8.2%), followed by Meseret, 3/85 (3.5%); Atsebekafa, 2/85 (2.4%); and Felege Abyot, 1/85 (1.2%) (Figure 1). Among rural elementary school children, high rate of* S. aureus* was observed in Kelel Rufael, 20/279 (7.2%), followed by Maryam Deber, 17/279 (6.1%); Arbaba, 12/279 (4.3%); Walaji, 8/279 (2.9%); and Azezo Tekelhaymanot, 1/279 (0.4%). But only one MRSA strain was found in Kelel Rufael, 1/58 (1.7%), of rural elementary school (Figure 2).

### 3.3. Antimicrobial Susceptibility Patterns

The antimicrobial susceptibility pattern result showed that, among 143* S. aureus* isolates, 129/143 (90.2%) were MSSA and the rest, 14/143 (9.79%), were MRSA. Majority of the isolates of* S. aureus* were sensitive for gentamycin (98.6%), clindamycin (93.7%), ciprofloxacin (93%), cefoxitin (90.2%), cotrimoxazole (84%), and erythromycin (55%). Some isolates of* S. aureus*, 44/143 (30.8%), showed multidrug resistance patterns. However, majority of the isolates were resistant to penicillin (99.3%) and tetracycline (71.33%) (Table 3).

### 3.4. Risk Factors

Among 622 elementary school children included in the study, father's educational status (P = 0.039; AOR = 1.98; CI =1.063-3.685) and number of students in classroom belonged to 41-60 (P = 0.031; AOR = 0.41; CI = 0.18-0.92) and 21-40 category (P = 0.046; AOR = 0.38; CI = 0.15 -0.98) were significant risk factors for nasal colonization of* S. aureus*. However, majority of the study variables were not risk factors for nasal colonization of* S. aureus* (Table 4). Among the 143* S. aureus *nasal carriers of children in the study, living in urban of children (P = 0.036; AOR = 0.107; CI = 0.013- 0.866) was significantly associated with MRSA nasal colonization. However, majority of the study variables were not risk factors for MRSA nasal colonization (Table 5).

## 4. Discussion

According to studies which were conducted on nasal colonization of* Staphylococcus aureus*, approximately 20-30% of healthy persons are persistent nasal carriers with high colonization rates among children [3, 9]. There was an important association between nasal carriage rates of* S. aureus* and development of subsequent infections [6]. MRSA had emerged in various geographically distinct communities outside of health care settings without known health care associated risk factors [12].

In this study,* S. aureus* nasal carriage rate among school children was 23%. This finding of* S. aureus* nasal carriage was in line with the reports in Ghana (22.6%) [16]. However, higher prevalence of* S. aureus* nasal carriage rate was reported in this study than Serbia (2.59%), China (2.4%), Iraq (17.75%), Vietnam (10.4%), and Nigeria (18.3%) [2, 17–19]. On the other hand, this finding showed lower prevalence of* S. aureus *nasal carriage rate than the reports in Italy (39.2%), India (25%), Iran (28%), Iraq (30%), Nigeria (56.3%), Jimma (47.34%), and Bahir Dar (41%) [5, 20–25]. This variation of nasal carriage rate of* S. aureus* from other studies might be due to difference in the characteristics of the study population, quality of sampling, culturing techniques, geographical distribution, and diagnostic techniques.

The nasal carriage rate of MRSA among school children was 9.79%. This nasal carriage of MRSA was less than the WHO 2014 estimated range of MRSA for African region (12-80%) [26]. This finding of MRSA nasal carriage was in line with the reports of Iraq (11.1%) [23] and higher than the reports in Serbia (7.55%), Ghana (1.6%), Italy (1.54%), India (3.17%), and Jordan (7.1%) [16, 17, 20, 21, 27]. However, this MRSA nasal carriage was lower than the reports in Iran (21.74%), Nigeria (59.1%), Iraq (13.3%), Vietnam (12.3%), Jimma (18.8%), and Bahir Dar (13.8%) [18, 19, 22, 24, 25, 28]. This variation might be due to the difference in the diagnostic techniques which was used to confirm/detect MRSA other than cefoxitin disc detection method.

In this study, among 143 children who had* S. aureus*, 79/143(55.2%) were male which indicates higher proportion of nasal carriage rate of* S. aureus* among male students than females, 64/143 (44.8%). This result is in agreement with previous studies which were conducted in Nigeria and Iraq [1, 8]. This higher prevalence in males might be due to male students participated in sport activities more often than females which makes them more vulnerable to colonization. Nasal carriage of* S. aureus* and MRSA, 83/143 (58%) and 13/83(15.66%), respectively, was higher in urban than rural residents, 60/143(42%) and 1/60 (1.67%). This might be due to overcrowding and the presence of large number of governmental and nongovernmental health care facilities such as hospitals and health centers/clinics in the urban than rural community.

Regarding the antimicrobial susceptibility pattern result there were more than 99% and 70% resistance strains to penicillin and tetracycline, respectively. In this study antimicrobial susceptibility test showed that penicillin and tetracycline were the least effective agent with more than 70% bacterial resistance. This result of penicillin resistant was in line with various studies which was conducted on healthy children in China (87.5%), Nigeria (100%), Ghana (95%), Jimma (100%), and Bahir Dar (100%) [2, 16, 19, 24, 25]. Majority of* S. aureus* isolates showed 99.3% resistance to penicillin. Similarly, MRSA isolates showed 100% resistance to penicillin and cefoxitin. This result was similar to a study conducted in Nigeria (100%), Jimma (100%), and Bahir Dar (100%) [1, 24, 25].

Majority of the isolated* S. aureus* were sensitive for clindamycin (94%), gentamycin (98.6%), cefoxitin (90.2%), cotrimoxazole (84%), and ciprofloxacin (93%). However, some of them, 32/143(22.4%), were intermediate resistance for erythromycin. This finding was similar to several studies in Serbia, Jimma, Jordan, and Bahir Dar [17, 24, 25, 27]. Gentamycin was the most susceptible antibiotic (98.6%) which was in agreement with a study in Nigeria (95%) [1].

The majority of the isolated* S. aureus* strains were sensitive for tested antimicrobial agents. However, 44/143 (30.8%) of isolates of* S. aureus *showed multidrug resistance patterns. The highest MDR pattern, 27/143 (18.9%), was observed for penicillin, tetracycline, and erythromycin. 5/143 (3.5%) of isolates were resistant for penicillin, cotrimoxazole, tetracycline, and erythromycin. One isolate was resistant for six antibiotics: penicillin, cotrimoxazole, tetracycline, erythromycin, clindamycin, and gentamycin. The differences in susceptibility pattern of the isolates might be due to frequent and irrational use of antibiotics, illegal drug healing in our setup, and differences in geographical area.

Among elementary school children, high rate of isolation of MRSA was reported in urban elementary schools. The high prevalence of MRSA in urban schools than rural might be due to reasons; for example, urban schools were more closer to health care facility; high antibiotic selective pressure in urban settings and overcrowding was more prominent in urban than rural schools.

Several studies showed that sociodemographic characteristics and risk factors contribute a lot to the nasal carriage of* S. aureus* and MRSA [5, 21, 22, 24, 25, 27, 28]. The result of this study showed children's father's educational status significantly associated with nasal carriage of* S. aureus* which was in contrast with a study in Nigeria [12]. The less the level of education among fathers of school children, the more likely for nasal carriage of* S. aureus *was observed. The possible reason might be that the less level of the fathers education, the less they care their children socioeconomically and hygienically.

Number of students in classroom was significant risk factors for nasal colonization of* S. aureus*. Majority of the students who had high nasal carriage of* S. aureus* belonged among children with 41-60 classmates in the class room which showed that the increase in the number of students per class room, the more likely the nasal carriage of* S. aureus* was observed in school children. This result was consistent with study conducted in Ethiopia, Jimma [24]. The possible reason might be due to the fact that high number of students in one class room makes them more frequent contact among each other, overcrowding, and greater sharing of nasal flora which causes the more spread of the bacteria.

Living in urban of children involved in this study was statistically significant risk factor for nasal colonization of MRSA. This study showed that being urban elementary school children had more likely hood for nasal carriage of MRSA than rural school children. The possible reason might be due to children living in the urban community which makes them contact with health care workers more frequently and more exposed to health care facilities and overcrowding in the urban area which increases nasal carriage of MRSA in urban schools.

## 5. Limitation of the Study

We did not perform vancomycin minimum inhibitory concentration due to resource constraints and advanced molecular techniques like PCR which may underestimate the true prevalence in the study population.

## 6. Conclusion

The prevalence of* S. aureus* and MRSA among primary school children was 23% and 9.79%, respectively. Father's educational status and number of students in class room were independent risk factors for nasal colonization of* S. aureus*. Moreover, being urban school children's were at risk of MRSA nasal colonization. Therefore, health education, screening of target population, and decolonization of carriers are effective prevention strategies to control the spread and burden of drug resistant* S. aureus* and MRSA within elementary schools and community. Large community-based studies, molecular distinction between hospital acquired-MRSA and community acquired-MRSA, continuous surveillance, screening of target population, and decolonization of carriers should be conducted to decrease the spread and burden of drug resistant* S. aureus* and MRSA in the elementary schools and the community at large.

## Figures and Tables

**Table 1:** Sociodemographic characteristics of study participants among elementary school children in Gondar, Northwest Ethiopia, 2018.

**Characteristics**		**Frequency ( n = 622)**	**Percentage (**%**)**
**Residence**	Urban	345	55.5
	Rural	277	44.5

**Sex**	Male	317	51
	Female	305	49

**Age in years**	6 -10 years	221	35.5
	10 -15 years	345	55.5
	> 15 years	56	9.0

**Educational status**	Grade 1-4	308	49.5
	Grade 5-8	314	50.5

**Family's monthly income**	Less than 500 birr	295	47.4
	500-1000 birr	130	20.9
	Greater than 1000 birr	197	31.7

**Family size category**	2-5	301	48.4
	Greater than 5	321	51.6

**Number of students in classroom category**	21-40	75	12.1
	41-60	486	78.1
	> 60	61	9.8

**History of chronic disease**	Yes	77	12.4
	No	545	87.6

**History of hospitalization**	Yes	100	16.1
	No	522	83.9

**History of surgery**	Yes	39	6.3
	No	583	93.7

**History of visit hospitals/clinics**	Yes	299	48.1
	No	323	51.9

**History of contact with health care worker**	Yes	71	11.4
	No	551	88.6

**History of antibiotic usage in the past 4 weeks**	Yes	93	15.0
	No	529	85.0

**History of respiratory infection**	Yes	91	14.6
	No	531	85.4

**Mothers' educational status**	Unable to read & write	271	43.6
	Informal education	68	10.9
	Primary school	153	24.6
	Secondary school	81	13.0
	Above grade 12	49	7.9

**Fathers' educational status**	Unable to read & write	211	33.9
	Informal education	97	15.6
	Primary school	146	23.5
	Secondary school	103	16.6
	Above grade 12	65	10.5

**Table 2:** Prevalence of *Staphylococcus aureus* with special emphasis on MRSA among study participants in urban and rural elementary school children in Gondar, Northwest Ethiopia, 2018.

**Characteristics**		**No. (**%**)**	**Schools**					
			**Urban**			**Rural**		
			**+ Ve for S.A (**%**)**	**+ Ve for MRSA (**%**)**	**-ve for S.A (**%**)**	** +ve for S.A (**%**)**	**+ Ve for MRSA (**%**)**	**-ve for S.A (**%**)**
**Age**	6-10 years	221 (35.53)	30 (22.73)	7 (23.33)	102 (77.27)	18 (20.23)	1 (5.56)	71 (79.78)
	11-15 years	345 (55.47)	50 (26.32)	4 (8)	140 (73.68)	31 (20)	0 (0)	124 (80)
	> 15 years	56 (9.0)	3 (13.04)	2 (8.70)	20 (86.96)	11 (33.33)	0 (0)	22 (66.67)
	Total	622 (100)	83 (24.06)	13 (15.66)	262 (75.94)	60 (21.66)	1 (1.67)	217 (78.34)

**Sex**	Male	317 (50.97)	45 (25.57)	6 (13.33)	128 (72.73)	34 (23.61)	1 (2.94)	110 (76.39)
	Female	305 (49.04)	38 (22.09)	7 (18.42)	134 (77.91)	26 (19.55)	0 (0 )	107 (80.45)
	Total	622 (100)	83 (24.06)	13 (15.66)	262 (75.94)	60 (21.66)	1(1.67)	217 (78.34)

**Family size**	2-5	301 (48.39)	44 (22.45)	8 (18.18)	152 (77.55)	18 (17.14)	0 (0)	87 (82.86)
	> 5	321 (51.61)	39 (26.17)	5 (12.82)	110 (73.83)	42 (24.42)	1 (2.38)	130 (75.58)
	Total	622 (100)	83 (24.06)	13 (15.66)	262 (75.94)	60 (21.66)	1 (1.67)	217 (78.34)

**Residence**		622 (100)	83 (24.06)	13 (15.66)	262 (75.94)	60 (21.66)	1 (2.67)	217 (78.34)

**Educational level**	Grade 1	74 (11.9)	9 (23.68)	1(11.11)	29 (76.32)	8 (22.22)	0 (0)	28 (77.78)
	Grade 2	75 (12.06)	8 (18.18)	1 (12.5)	36 (81.81)	8 (25.81)	1 (12.5)	23 (74.19)
	Grade 3	79 (12.7)	7 (18.42)	2 (28.57)	31 (81.58)	5 (122)	0 (0)	36 (87.81)
	Grade 4	78 (12.54)	16 (37.21)	2 (12.5)	27 (62.79)	10 (28.57)	0 (0)	25 (71.43)
	Grade 5	87 (13.99)	11 (21.15)	3 (27.27)	41 (78.85)	6 (17.14)	0 (0)	29 (82.86)
	Grade 6	81 (13.02)	11 (26.19)	1 (9.09)	31 (73.81)	6 (15.39)	0 (0)	33 (84.62)
	Grade 7	77 (12.38)	11 (26.19)	0 (0)	31 (73.81)	10 (28.57)	0 (0)	25 (71.43)
	Grade 8	71 (11.42)	10 (21.74)	3 (30)	36 (78.26)	7 (28)	0 (0)	18 (72)
	Total	622 (100)	83 (24.06)	13 (15.66)	262 (75.94)	60 (21.66)	1 (1.67)	217 (78.34)

**Table 3:** Antibiotic susceptibility patterns of *Staphylococcus aureus* isolates in Gondar town, Northwest Ethiopia, 2018.

**Antibiotics**	**Schools**											
	**Urban (83)**			**Rural (60)**			**Urban**			**Rural**		
	**MSSA (70)**			**MSSA (59)**			**MRSA (13)**			**MRSA (1)**		
	**S (**%**)**	**I (**%**)**	**R (**%**)**	**S (**%**)**	**I (**%**)**	**R (**%**)**	**S (**%**)**	**I (**%**)**	**R (**%**)**	**S (**%**)**	**I (**%**)**	**R (**%**)**
**Penicillin (10 **μ**g)**	0 (0)	0 (0)	70 (100)	1 (1.7)	0 (0 )	58 (98.31)	0 (0)	0 (0)	13 (100)	0 (0)	0 (0)	1 (100)
**Erythromycin (15 **μ**g)**	42 (60)	21 (30)	7 (10)	32 (54.4)	9 (15.3)	18 (30.5)	3 (23.08)	2 (15.39)	8 (61.54 )	1(100)	0 (0)	0 (0)
**Clindamycin (2 **μ**g)**	65 (92.9)	2 (2.88)	3 (4.29)	59 (100)	0 (0)	0 (0)	9 (69.23)	0 (0)	4 (30.77 )	1 (100)	0 (0)	0 (0)
**Gentamicin (10 **μ**g)**	69 (98.57)	0 (0)	1 (1.43)	59 (100)	0 (0)	0 (0)	12 (92.31)	1 (7.69)	0 (0 %)	1 (100 )	0 (0)	0 (0)
**TMP/SMX (1.25/23.75 **μ**g)**	59 (84.29)	6 (8.57)	5 (7.14)	51 (86.44)	2 (3.39)	6 (10.17)	9 (69.23)	1 (7.69)	3 (23.08)	1 (100)	0 (0)	0 (0)
**Cefoxitin (30 **μ**g)**	70 (100)	0 (0)	0 (0)	59 (100)	0 (0)	0 (0)	0 (0)	0 (0)	13 (100)	0 (0 )	0 (0)	1 (100)
**Ciprofloxacin (30 **μ**g)**	63 (90)	6 (8.57)	1 (1.43)	59 (100)	0 (0)	0 (0)	10 (76.92)	1 (7.69)	2 (15.38)	1 (100)	0 (0)	0 (0)
**Tetracycline (30 **μ**g)**	11 (15.71)	14 (20)	45 (64.29)	7 (11.86)	4 (6.78)	48 (81.67)	1 (7.36)	4 (30.77)	8 (61.54)	0 (0)	0 (0)	1 (100)

S= susceptible, I = intermediate, and R = resistance.

**Table 4:** Epidemiologic risk factors for *Staphylococcus aureus* colonization among elementary school children in Gondar town, Northwest Ethiopia, 2018.

**Characteristics**		**Number (**%**)**	**Overall *Staphylococcus aureus *isolates**					
			**No. (**%**) positive for *S. aureus***	**No. (**%**) negative for *S. aureus***	**Crude OR (95**%** CI)**	**P value**	**Adjusted OR (95**%** CI)**	**P value**
**Residence**	Urban	345 (55.47)	83 (24.06)	262 (75.94)	0.87 [0.6-1.27]	0.48	-	-
	Rural	277 (44.53)	60 (21.66)	217 (78.34)	1(ref)	1(ref)	-	-

**Sex**	Male	317 (51)	79 (24.92)	238 (75.08)	0.8 [0.55-1.16]	0.244	-	-
	Female	305 (49)	64 (20.98)	241(79.02)	1(ref)	1(ref)	-	-

**Age in years**	6-10 years	221 (35.5)	48 (21.72)	173 (78.28)	1.20 [0.606-2.381]	0.599	-	-
	10-15 years	345 (55.5)	81 (23.48)	264 (76.52)	1.09 [0.565-2.090]	0.804	-	-
	> 15 years	56 (9.0)	14 (25)	42 (75)	1(ref)	1(ref)	-	-

**Educational status**	Grade 1-4	308 (49.5)	71 (23.05)	237 (76.95)	0.99 [0.684-1.443]	0.971	-	-
	Grade 5-8	314 (50.5)	72 (22.93)	242 (77.07)	1(ref)	1(ref)	-	-

**Family's monthly income**	<500 birr	295 (47.4)	59 (20)	236 (80)	1.51 [0.989-2.307]	0.056	1.38 [0.83-2.27]	0.214
	500-1000 birr	130 (20.9)	30 (23.08)	100 (76.92)	1.26 [0.753-2.105]	0.380	1.13 [0.65-1.97]	0.66
	>1000 birr	197 (31.7)	54 (27.41)	143 (72.59)	1(ref)	1(ref)	1(ref)	1(ref)

**Family size category**	2-5	301 (48.4)	62 (20.6)	239 (79.40)	1.30 [0.893-1.895]	0.17	1.33 [0.90-1.96]	0.148
	>5	321 (51.6)	81 (25.23)	24 0 (74.77)	1(ref)	1(ref)	1(ref)	1(ref)

**No. of students in classroom category**	21-40	75 (12.06)	19 (25.3)	56 (74.7)	0.38 [0.15-0.98]	0.046	0.38 [0.15-0.98]	0.046
	41-60	486 (78.14)	117 (24.1)	369 (75.9)	0.41 (0.18-0.9]	0.031	0.41 [0.18-0.92]	0.031
	>60	61 (9.8)	7 (11.5)	54 (88.5)	1(ref)	1(ref)	1(ref)	1(ref)

**History of chronic disease**	Yes	77 (12.4)	18 (23.38)	59 (76.62)	1.03 [0.58-1.80]	0.931	-	-
	No	545 (87.6)	125 (22.94)	420 (77.06)	1(ref)	1(ref)	-	-

**History of hospitalization**	Yes	100 (16.1)	23 (23)	77 (77)	1.00 [0.60-1.66]	0.998	-	-
	No	522 (83.9)	120 (22.99)	402 (77.01)	1(ref)	1(ref)	-	-

**History of surgery**	Yes	39 (6.3)	9 (23)	30 (77)	1.01 [0.47-2.17]	0.989	-	-
	No	583 (93.7)	134 (21.27)	449 (77.02)	1(ref)	1(ref)	-	-

**History of visit hospitals/clinic**	Yes	299 (48.1)	71 (23.75)	228 (76.25)	1.09 [0.747-1.577]	0.667	-	-
	No	323 (51.9)	72 (22.29)	251 (77.71)	1(ref)	1(ref)	-	-

**History of contact with health care worker**	Yes	71 (11.4)	18 (25.35)	53 (74.65)	1.16 [0.654-2.048]	0.616	-	-
	No	551 (88.6)	125 (22.69)	426 (77.31)	1(ref)	1(ref)	-	-

**History of antibiotic usage in the past 4 weeks**	Yes	93 (15.0)	20 (21.51)	73 (78.5)	0.90 [0.53-1.54]	0.712	-	-
	No	529 (85.0)	123 (23.21)	406 (76.75)	1(ref)	1(ref)	-	-

**Adherence to antibiotic therapy**	Completed	73 (11.7)	13 (17.81)	60 (82.19)	1.406 [0.75-2.65]	0.291	-	-
	Not completed	18 (2.9)	6 (33.33)	12 (66.67)	1(ref)	1(ref)	-	-

**History of respiratory infection**	Yes	91 (14.6)	22 (24.18)	69 (75.82)	1.08 [0.64-1.82]	0.771	-	-
	No	531 (85.4)	121 (22.79)	410 (77.21)	1(ref)	1(ref)	-	-

**Mothers' educational status**	Unable to read & write	271 (43.6)	57(21.033)	214 (78.97)	1.2 [1.03-3.85]	0.039	1.57 [0.723-3.422]	0.253
	Informal education	68 (10.9)	17 (25)	51 (75)	1.594 [0.713-3.563]	0.256	1.49 [0.596-3.733]	0.371
	Primary school	153 (24.6)	35 (22.88)	118 (77.12)	1.79 [0.890-3.602]	0.102	1.47 [0.678-3.190]	0.299
	Secondary school	81 (13.0)	17 (20.99)	64 (79.01)	2.0 [0.903-4.429]	0.087	1.68 [0.714-3.970]	0.217
	Above grade 12	49 (7.9)	17 (34.69)	32 (65.30)	1(ref)	1(ref)	1(ref)	1(ref)

**Fathers' educational status**	Unable to read & write	211 (33.9)	41 (19.43)	170 (80.57)	1.98 [1.063-3.685]	0.031	1.98 [1.063-3.685]	0.039
	Informal education	97 (15.6)	28 (28.87)	69 (71.13)	1.18 [0.596-2.323]	0.640	1.18 [0.596-2.323]	0.715
	Primary school	146 (23.5)	31 (21.23)	115 (78.77)	1.77 [0.921-3.405]	0.087	1.77 [0.921-3.405]	0.105
	Secondary school	103 (16.6)	22 (21.36)	81 (78.64)	1.76 [0.871-3.544]	0.115	1.78 [0.871-3.544]	0.132
	Above grade 12	65 (10.5)	21 (32.31)	44 (67.69)	1(ref)	1(ref)	1(ref)	1(ref)

**Note**: ref: reference; AOR: adjusted odds ratio; CI: confidence interval; OR: odds ratio.

**Table 5:** Risk factors for MRSA nasal colonization among elementary school children in Gondar town, Northwest Ethiopia, 2018.

**Characteristics**		**No. (**%**) +ve for *S. aureus***	**Methicillin resistant *Staphylococcus aureus* (MRSA)**					
			**No. (**%**) positive for MRSA **	**No. (**%**) negative for MRSA**	**Crude OR (95**%** CI)**	**P value**	**Adjusted OR (95**%** CI)**	**P value**
**Residence**	Urban	83 (58.04)	13 (15.66)	70 (84.34)	0.09 [0 .012- 0.718]	0.023	0.107 [0.013-0.866]	0.036
	Rural	60 (41.96)	1 (1.67)	59 (98.33)	1(ref)	1(ref)	1(ref)	1(ref)

**Sex **	Male	79 (55.2)	7 (8.86)	72 (91.14)	1.26 [0.419-3.809]	0.678	-	-
	Female	64 (44.8)	7 (10.94)	57 (89.06)	1(ref)	1(ref)	-	-

**Age in years**	6-10 years	48 (33.6)	8 (16.67)	40 (83.33)	0.83 [0.156-4.46]	0.831	-	-
	10-15 years	81 (56.6)	4 (4.94)	77 (95.06)	3.21 [0.529-19.47]	0.205	-	-
	> 15 years	14 (9.8)	2 (14.29)	12 (85.714)	1(ref)	1(ref)	-	-

**Educational status**	Grade 1-4	71 (49.65)	7 (9.86)	64 (90.14)	0.99 [0.327-2.967]	0.978	-	-
	Grade 5-8	72 (50.35)	7 (9.72)	65 (90.28)	1(ref)	1(ref)	-	-

**Family's average monthly income**	Less than 500 birr	59 (41.26)	4 (6.78)	55 (93.22)	1.72 [0.458-6.454]	0.422	-	-
	500-1000 birr	30 (20.98)	4 (13.33)	26 (86.67)	0.81 [0.210-3.141]	0.763	-	-
	>1000 birr	54 (37.76)	6 (11.11)	48 (88.89)	1(ref)	1(ref)	-	-

**Family size category**	2-5	62 (43.4)	8 (12.90)	54 (87.1)	0.54 [0.177-1.646]	0.279	-	-
	>5	81 (56.6)	6 (7.41)	75 (92.59)	1(ref)	1(ref)	-	-

**History of chronic disease**	Yes	18 (12.59)	4 (22.22)	14 (77.78)	3.29 [0.909-11.882]	0.07	2.4 [0.51- 8.18]	0.313
	No	125 (87.41)	10 (8)	115 (92)	1(ref)	1(ref)	1(ref)	1(ref)

**History of hospitalization **	Yes	23 (16.1)	4 (17.91)	19 (82.61)	2.32 [0.658-8.145]	0.191	1.29 [0.34- 4.94]	0.71
	No	120 (83.9 )	10 (8.33)	110 (91.67)	1(ref)	1(ref)	1(ref)	1(ref)

**History of surgery**	Yes	9 (6.3)	2 (22.22)	7 (77.78)	2.905 [0.542-15.58]	0. 213	-	-
	No	134 (93.7)	12 (8.96)	122 (91.05)	1(ref)	1(ref)	-	-

**History of visit hospitals/clinics **	Yes	71 (49.7)	8 (11.27)	63 (88.72)	1.4 [0.459-4.253]	0.556	-	-
	No	72 (50.3)	6 (8.333)	66 (91.67)	1(ref)	1(ref)	-	-

**History contact with health care worker**	Yes	18 (12.6)	3 (16.67)	15 (83.33)	2.07 [0.519-8.284]	0.302	-	-
	No	125 (87.4)	11 (8.8)	114 (91.2)	1(ref)	1(ref)	-	-

**History of antibiotic usage in the past 4 weeks**	Yes	20 (14.0)	4 (20)	16 (80)	2.83 [0.792-10.082]	0.11	2.66 [0.71- 10]	0.149
	No	123 (86.0)	10 (8.13)	113 (91.87)	1(ref)	1(ref)	1(ref)	1(ref)

**Adherence to antibiotic therapy**	Completed	13 (65)	2 (15.85)	11 (84.62)	0.48 [0.094-2.486]	0.384	-	-
	Not completed	7 (35)	2 (28.57)	5 (71.43)	1(ref)	1(ref)	-	-

**History of respiratory infection**	Yes	22 (15.39)	1 (4.55)	21 (95.45)	0.4 [0.049-3.189]	0.384	-	-
	No	121 (84.62)	13 (10.74)	108 (89.26)	1(ref)	1(ref)	-	-

**Mothers' educational status**	Unable to read & write	57 (39.9)	3 (5.26)	54 (94.74)	3.86 [0.701-21.216]	0.121	1.49 [0.22- 10.1]	0.683
	Informal education	17 (11.9)	3 (17.65)	14 (82.35)	1.00 [0.171-5.833]	1.00	0.66 [0.101-4.24]	0.657
	Primary school	35 (24.5)	3 (8.57)	32 (91.43)	2.29 [0.410-12.754]	0.346	1.14 [0.166-7.88]	0.891
	Secondary school	17 (11.9)	2 (11.77)	15 (88.24)	1.61 [0.233-11.092]	0.63	1.124 [0.14- 9.00]	0.912
	Above grade 12	17 (11.9)	3 (17.65)	14 (82.35)	1(ref)	1(ref)	1(ref)	1(ref)

**Fathers' educational status**	Unable to read & write	41 (28.7)	2 (4.88)	39 (95.12)	3.25 [0.499-21.179]	0.218	-	-
	Informal education	28 (19.6)	2 (7.14)	26 (92.86)	2.17 [0.328-14.305]	0.422	-	-
	Primary school	31 (21.7)	4 (12.9)	27 (87.1)	1.13 [0.225-5.636]	0.886	-	-
	Secondary school	22 (15.4)	3 (13.64)	19 (86.36)	1.06 [0.188-5.926]	0.951	-	-
	Above grade 12	21 (14.7)	3 (14.29)	18 (85.71)	1(ref)	1(ref)	-	-

**Note**: ref: reference; AOR: adjusted odds ratio; CI: confidence interval; OR: odds ratio.

## Data Availability

All data generated or analyzed during this study are included in this article. Data that support the findings of this study are also available from the corresponding author upon reasonable request.

## Abbreviations

ATCC: American Type of Culture Collection
CI: Confidence Interval
CLSI: Clinical and Laboratory Standards Institute
EPI-Info: Epidemiological Information
MRSA: Methicillin Resistance* Staphylococcus aureus*
MSSA: Methicillin Susceptible* Staphylococcus aureus*
AOR: Adjusted odds ratio
PBP2a: Penicillin binding protein
S.A: * Staphylococcus aureus*
*S. aureus*: * Staphylococcus aureus*
SCCmec: Staphylococcal Cassette Chromosome mec
SD: Standard Deviation
SPSS: Statistical Package for Social Sciences
TMP/SMZ: Trimethoprim-sulfamethoxazole
TSY: Tryptone Soya Broth
WHO: World Health Organization.

## References

[1] Ugwu M., Mokwe N., Ejikeugwu P. (2015). Antibiogram of Staphylococcus aureus from healthy school pupils in Agulu, Southeastern Nigeria. *International Journal of Research in Pharmacy and Biosciences*.

[2] Deng J., Xiao G., Zhu Y., Zhou W., Wan C. (2014). Staphylococcus aureus nasal carriage and its antibiotic resistance profiles in Tibetan school children in Southwest China. *Hong Kong Journal of Pediatrics*.

[3] Paulino C., Garcia R. D., Ong S. (2013). Staphylococcus aureus nasal carriage rates among children between one-to-five years in Barangay Pio Del Pilar, Makati City. *Pediatric Infectious Disease Society of the Philippines Journal*.

[4] Miller M. B., Weber D. J., Goodrich J. S. (2011). Prevalence and risk factor analysis for methicillin-resistant Staphylococcus aureus nasal colonization in children attending child care centers. *Journal of Clinical Microbiology*.

[5] Nsofor C., Nwokenkwo V., Nwaokpa C. (2015). Nasal Carriage of Staphylococcus Aureus among Apparently Healthy School Children in Owerri Metropolis, Nigeria. *MOJ Cell Science & Report*.

[6] Campoccia D., Montanaro L., Arciola C. R. (2006). The significance of infection related to orthopedic devices and issues of antibiotic resistance. *Biomaterials*.

[7] Rudkin J. K., Laabei M., Edwards A. M. (2014). Oxacillin alters the toxin expression profile of community-associated methicillin-resistant Staphylococcus aureus. *Antimicrobial Agents and Chemotherapy*.

[8] Assafi M. S., Mohammed R. Q., Hussein N. R. (2015). Nasal carriage rates of *Staphylococcus aureus* and Community associated-methicillin resistant *Staphylococcus aureus* among university students. *Journal of Microbiology Research*.

[9] Davoodabadi F., Mobasherizadeh S., Mostafavizadeh K. (2016). Nasal colonization in children with community acquired methicillin-resistant Staphylococcus aureus. *Advanced Biomedical Research*.

[10] Ansari S., Gautam R., Shrestha S., Ansari S. R., Subedi S. N., Chhetri M. R. (2016). Risk factors assessment for nasal colonization of Staphylococcus aureus and its methicillin resistant strains among pre-clinical medical students of Nepal. *BMC Research Notes*.

[11] Raygada J. L., Levine D. P. (2009). Managing CA-MRSA infections: Current and emerging options. *Infections in Medicine*.

[12] Srinivasan A., Seifried S. E., Zhu L. (2010). Increasing prevalence of nasal and rectal colonization with methicillin-resistant Staphylococcus aureus in children with cancer. *Pediatric Blood & Cancer*.

[13] Maree C. L., Eells S. J., Tan J. (2010). Risk factors for infection and colonization with community-associated methicillin-resistant Staphylococcus aureus in the Los Angeles County jail: A case-control study. *Clinical Infectious Diseases*.

[14] Sobhy N., Aly F., El Kader O. A., Ghazal A., Elbaradei A. (2012). Community-acquired methicillin-resistant Staphylococcus aureus from skin and soft tissue infections (in a sample of Egyptian population): Analysis of mec gene and staphylococcal cassette chromosome. *The Brazilian Journal of Infectious Diseases*.

[15] Wayne P. A., CLSI (2017). *Performance standards for antimicrobial susceptibility testing*.

[16] Eibach D., Nagel M., Hogan B. (2017). Nasal carriage of staphylococcus aureus among children in the Ashanti region of Ghana. *PLoS ONE*.

[17] Dinić M., Vuković S., Kocić B., Dordević D. S., Bogdanović M. (2013). Nasal carriage of Staphylococcus aureus in healthy adults and in school children. *Acta Facultatis Medicae Naissensis*.

[18] Hussein N. R., Basharat Z., Muhammed A. H., Al-Dabbagh S. A. (2015). Comparative evaluation of MRSA nasal colonization epidemiology in the Urban and Rural Secondary School Community of Kurdistan, Iraq. *PLoS ONE*.

[19] Okwu M., Bamgbala S., Aborisade W. (2012). Prevalence of nasal carriage of community-associated Methicillin-resistant *Staphylococcus aureus* (CA-MRSA) among healthy primary school children in Okada, Nigeria. *Prevalence*.

[20] Esposito S., Terranova L., Zampiero A. (2014). Oropharyngeal and nasal carriage by healthy children. *BMC Infectious Diseases*.

[21] Shetty V., Trumbull K., Hegde A. (2014). Prevalence of community-acquired methicillin-resistant staphylococcus aureus nasal colonization among children. *Journal of Clinical and Diagnostic Research*.

[22] Mobasherizadeh S., Shojaei H., Havaei S. (2016). Nasal carriage screening of community-associated methicillin resistant Staphylococcus aureus in healthy children of a developing country. *Advanced Biomedical Research*.

[23] Assafi M., Polse R., Hussein N., Haji A., Issa A. (2017). The Prevalence of S. Aureus Nasal Colonisation and its Antibiotic Sensitivity Pattern amongst Primary School Pupils. *Science Journal of University of Zakho*.

[24] Kejela T., Bacha K. (2013). Prevalence and antibiotic susceptibility pattern of methicillin-resistant Staphylococcus aureus (MRSA) among primary school children and prisoners in Jimma Town, Southwest Ethiopia. *Annals of Clinical Microbiology and Antimicrobials*.

[25] Reta A., Gedefaw L., Sewunet T., Beyene G. (2015). Nasal carriage, risk factors and antimicrobial susceptibility pattern of methicillin resistant *Staphylococcus aureus* among school children in Ethiopia. *Journal of Medical Microbiology & Diagnosis*.

[26] Organization WH (2014). *Antimicrobial resistance: global report on surveillance*.

[27] Alzoubi H. M., Aqel A. A., Al-Sarayreh S. A., Al-Zayadneh E. (2014). Methicillin-resistant Staphylococcus aureus nasal carriage among primary school-aged children from Jordan: Prevalence, antibiotic resistance and molecular characteristics. *Journal of the Egyptian Public Health Association*.

[28] Van Nguyen K., Zhang T., Vu B. N. T. (2014). Staphylococcus aureus nasopharyngeal carriage in rural and urban northern Vietnam. *Transactions of the Royal Society of Tropical Medicine and Hygiene*.

